# 'Pragmatic randomized controlled trial of individually prescribed exercise versus usual care in a heterogeneous cancer survivor population': A feasibility study PEACH Trial: Prescribed exercise after chemotherapy

**DOI:** 10.1186/1471-2407-10-42

**Published:** 2010-02-15

**Authors:** Julie M Walsh, Juliette Hussey, Emer Guinan, Dearbhaile O' Donnell

**Affiliations:** 1 Discipline of Physiotherapy, Trinity Centre for Health Sciences, St James's Hospital, Dublin 8, Ireland; 2 Department of Medical Oncology, St James's Hospital, Dublin 8, Ireland

## Abstract

**Background:**

Many cancer survivors suffer a range of physical and psychological symptoms which may persist for months or years after cessation of treatment. Despite the known benefits of exercise and its potential to address many of the adverse effects of treatment, the role of exercise as well as optimum duration, frequency, and intensity in this population has yet to be fully elucidated. Many cancer rehabilitation programmes presented in the literature are very long and have tight eligibility criteria which make them non-applicable to the majority of cancer survivors. This paper presents the protocol of a novel 8-week intervention which aims to increase fitness, and address other physical symptoms in a heterogeneous cancer survivor population.

**Methods/design:**

The aim is to recruit 64 cancer survivors 2-6 months after completion of chemotherapy, usually adjuvant, with curative intent. Subjects will be recruited through oncology clinics in a single institution and randomised to usual care or an exercise intervention. The exercise intervention consists of two specifically tailored supervised moderate intensity aerobic exercise sessions weekly over 8-weeks. All participants will be assessed at baseline (0 weeks), at the end of the intervention (8 weeks), and at 3-month follow-up. The primary outcome measure is fitness, and secondary patient-related outcome measures include fatigue, quality of life, and morphological outcomes. A further secondary outcome is process evaluation including adherence to and compliance with the exercise program.

**Discussion:**

This study will provide valuable information about the physical outcomes of this 8-week supervised aerobic programme. Additionally, process information and economic evaluation will inform the feasibility of implementing this program in a heterogeneous population post cessation of chemotherapy.

**Trial Registration:**

NCT01030887

## Background

The benefits of exercise in the general population are well established [1,2]. The current physical activity recommendation for adults aged between 18-65 years to promote and maintain health is to accumulate at least 30 minutes of moderately intense physical activity on at least five days of the week. Sedentary behaviour, such as time spent sitting is positively associated with coronary heart disease risk factors, obesity, and development of the metabolic syndrome [3,4]. More recently, the link between sedentary behaviour and the development of certain cancer types has been reported.

Newer evidence indicates that increased physical activity after a cancer diagnosis may reduce the risk of cancer recurrence and mortality [5-7]. Cancer survivors are at risk of developing new primary tumours as well as secondary tumours and chronic diseases such as cardiovascular disease, osteoporosis, and diabetes [8]. This increased risk across conditions may be genetic or treatment-related but may also reflect the fact that common life-style factors, including a sedentary lifestyle, increase the risk for more than one disease [8].

Physical activity is also advocated during and after the treatment for cancer. However, the adverse effects of treatment which frequently impact on physical functioning and quality of life, may persist even years after treatment [9-11], and must be taken into account. Many treatments for cancer are toxic, increasing the risk for a number of medical problems and late treatment effects such as neuropathy [12] and cardiovascular disease [12-14]. Cardiopulmonary capacity may also be compromised in cancer survivors because of side effects of therapy regimens such as weight gain and inactivity secondary to treatment. The reduction in cardiopulmonary capacity may lead to decreases in quality of life [15,16]. A downward trajectory in aerobic fitness is generally observed, possibly caused by chemotherapy and associated side-effects such as anaemia, tachycardia, dehydration, and cardiac dysfunction [17,18].

Other cancer survivors experience somatic symptoms which may also persist for years after treatment such as chronic fatigue [19,20], weight gain [19], loss of muscle strength [21,22] and difficulty sleeping [20]. Physical and functional well-being are essential dimensions for overall quality of life [23], and poor physical functioning may explain some of the psychological distress experienced by cancer survivors [21,22].

The rationale for physical activity interventions following cancer diagnosis includes minimizing biological processes associated with tumour growth [24], enhancing behavioural changes to try to minimise lifestyle risk factors for recurrence of cancer [25], and improving psychosocial factors during and after cancer [26]. The main physiological outcomes of physical activity after cancer are improved fitness and physical functioning [27,28], reduced fatigue [29], and modestly decreased weight and body fat [30].

Even though there is strong evidence for the benefits of physical activity aftercancer-treatment, the majority of cancer survivors do not meet public health guidelines for levels of physical activity necessary for health-related benefits [31-34]. Clearly, there is a need to promote physical activity, but the optimal intervention modality, intensity, training and duration are still unknown.

Many programmes in cancer rehabilitation are mainly based on psychotherapy or social support. Such therapies however do not usually deal with the physical problems encountered by many patients, such as fatigue, loss of functional capacity and weight gain [35]. Many cancer rehabilitation programs and/or trials which include a physical rehabilitation are lengthy in duration, extremely intensive [36] and have tight eligibility criteria. They would be expensive to translate into general clinical practice or to apply to the general cancer survivor population.

We have therefore devised a RCT to evaluate the effectiveness and feasibility of an 8-week intervention to improve fitness and other secondary outcomes.

## Methods/Design

### Study design

This will be a single-blind 2 arm pragmatic randomised controlled trial of individually prescribed exercise in a class setting versus usual care.

### Primary aims

#### Outcome evaluation

To examine the effects of a supervised exercise program commenced within 2-6 months after completion of chemotherapy and/or radiotherapy with curative intent on cardio-respiratory fitness, and on quality-of-life, fatigue and morphological outcomes.

#### Process Evaluation

As this is a feasibility study, an additional aim is to determine the acceptability of the programme, as well as adherence and compliance with the prescribed duration and intensity during supervised exercise sessions.

### Patient Recruitment

Participants will be sourced from the institutional cancer database, by invitation from study personnel in cancer clinics and directly from the oncology service in St. James's Hospital, Dublin, Ireland. Study personnel will provide further information to these patients and assess them for eligibility. Ethical approval has been approved from the institutional Research Ethics Committee and informed consent will be gained from each participant prior to study inclusion.

Aerobic fitness will be measured using the Modified Bruce treadmill test and those with aerobic fitness levels average or below age- and gender norms [37] and meeting other inclusion criteria will be invited to participate in the study. Subjects scoring average or below for age and gender cut-off levels according to the Modified Bruce Protocol, will be randomized to the exercise intervention or usual care.

### Inclusion criteria

(i) Diagnosis of solid tumour and completion of adjuvant chemotherapy or radiotherapy with curative intent within the preceding 2-6 months. Patients who have received neoadjuvant chemotherapy or chemoradiotherapy followed by surgery will also be eligible. Patients continuing onto adjuvant hormone therapy and anti-Her2 directed therapy are eligible

(ii) Ability to understand English

(iii) Willing to be randomised

(iv) Medical clearance to exercise

(v) Age 21-69

(vi) Fitness level average, fair or poor according to certain pre-determined cut-off points for age and gender[37]

(vii) Willing and able to attend supervised exercise sessions twice weekly for a period of 8 weeks, with an intention of achieving > 90% attendance

(viii) LVEF > 50% and not < 20% worse than baseline in applicable patients

### Exclusion criteria

(i) Evidence of active cancer

(ii) Chronic medical and orthopaedic conditions that would preclude exercise (eg uncontrolled congestive heart failure or angina, recent MI, breathing difficulties requiring oxygen use or hospitalization)

(iii) On beta-blocker medication

(iii) Prior history of another cancer in previous 5 years (exception: non-melanoma skin cancer and non-invasive cancer of the cervix)

(iv) Confirmed pregnancy

(v) Dementia or psychiatric illness that would preclude ability to participate in study

(vi) Incomplete haematological recovery after chemotherapy (WCC < 3, Hb < 10 or Platelets < 100)

(vii) BMI > 35

(viii) LVEF post chemotherapy < 50% or > 20% deterioration of baseline compared to LVEF before systemic treatment. LVEF criteria are applicable in patients who have received systemic therapy (eg anthracycles, Her-2 antagonists etc) deemed in normal clinical practice to have a potential effect LVEF and in whom the LVEF will have been measured before systemic treatment and at end of chemotherapy

### Assessment

Post randomization follow-up will be at baseline (week 0), end programme (8 weeks) and 3 month follow-up.

#### Baseline Characteristics

Cancer grade, stage, surgical management, treatment regimen (chemotherapy, radiation therapy, hormonal therapy) and previous medical history will be recorded by accessing medical charts. Socio-demographic variables recorded will include age, gender, ethnicity, employment status, smoking, and alcohol habits accessed from medical charts and/or subjective interview.

#### Body Composition

Anthropometric parameters will be measured at baseline, 8 weeks and at 3 months. Body weight will be measured to the nearest gram (g) using a standardised digital scales. Standing height will be measured, without shoes, to the nearest millimetre (mm) using stadiometer. BMI will be calculated by dividing weight in kilograms by height in metres squared.

Lean body mass will be evaluated using bioimpedence (Tanita). Waist Circumference will be measured as per the protocol outlined in the ACSM Resource Manual [38].

#### Cardiovascular Parameters

Resting heart rate will be measured after a five minute rest period in a seated position. Heart rate reserve will be calculated by subtracting the resting heart rate from peak heart rate during exercise. Oxygenation saturation will be monitored using a finger pulse oximeter.

Blood pressure (BP) will be measured using the auscultatory method in accordance with the Joint National Committee on Prevention, Detection, Evaluation, and Treatment of High Blood Pressure guidelines.

#### Outcome Measures

##### Primary Outcome measure: Fitness

Physical fitness will be measured by the Modified Bruce Protocol. A sub-maximal test was chosen over a maximal test for this population because of safety and motivational concerns [39]. Verbal encouragement during the exercise test will be standardised.

##### Secondary outcomes

##### Quality of life

We will use the FACT-G scale (general) (and the physical functional measure of the SF-36. Many uncontrollable factors influence QOL during and after chemotherapy and a global measure of cancer-specific QOL may be too broad to detect the likely narrower effects of exercise training, therefore a more appropriate and realistic primary end-point in exercise trials may be the functional component of QOL [40]. A statistically significant increase of greater than 4.0 points on the FACT scale represents a clinically meaningful improvement in quality of life from exercise [41].

The Fact-G (version 4) is a 27-item questionnaire divided into 4 primary QOL domains: physical well-being, social/family well-being, emotional wellbeing, and functional well-being and an overall quality of life level is also yielded. The FACT-G takes about 5 minutes to complete and has been written at the 6th grade level. This questionnaire was initially developed by Cella et al. 1993 and has been validated in a mixed cancer population [42]. Patients rate all items using a 5-point rating scale ranging from "not at all" to "very much". Strong concurrent validity is reported by strong Pearson correlations with the Functional Living Index - Cancer (0.79) and the patient-completed version of the QL index (0.74). Using the global rating of change (GRC) scale as an anchor, Cella et al proposed that a clinically meaningful change corresponds to a total FACT-G raw score in the range of 5-7 points [43]. A 2008 review by Victorson et al., reported the average reliability of the FACT-G to be 0.88 with the reliability of subsets ranging from 0.71-0.83.

The SF-36v2 is a widely used generic measure of health status. Psychometric properties of the SF-36 have been well established [44]. Thirty five of the 36 items are grouped into eight scales that address health constructs considered to be important to most health care situations: physical functioning, role limitations (physical problems), bodily pain, general health, vitality, social functioning, role limitations (emotional problems), and mental health We will use the Physical Health measure only of the SF-36 for the purposes of this study.

##### Current activity level

We will use the modified version of the Godin Leisure Time Exercise Questionnaire which has been shown to be a reliable and valid self-report measure of physical activity [45]. The questionnaire contains three questions that assess the average frequency and duration of mild, moderate, and strenuous exercise during free time in a typical week Godin et al. reported the test-retest reliability coefficient of the Godin questionnaire to be 0.64 and the concurrent validity to range from 0.38 to 0.54 over three validity criteria [45].

Accelerometry will be used to monitor 7 days of activity at Week 0, Week 8 (immediately post intervention) & 3 months post intervention. We will use the RT3 accelerometer (Stayhealthy Inc.) which has been shown to be reliable [46] and valid [47]. The RT3 accelerometer measures activity in three dimensions. With the RT3 worn on the hip, the vectors are as follows: vertical (x), antero-posterior (y), and medio-lateral (z) and generates a summary variable-vector magnitude.

##### Cancer-related fatigue

We will use the 13-item Functional Assessment of Chronic Illness Therapy Fatigue Scale (FACT-Fatigue) of the Functional Assessment of Chronic Illness Therapy measurement system which has been developed specifically for the cancer population [42,48]). The reliability of the FACT-fatigue scale has been reported to be 0.84 and it has been shown to have good validity with other measures of fatigue, a significant negative relationship with vigor and with social desirability [49].

##### Satisfaction

Self-administered questionnaire on completion of programme will be administered. Participants will also be asked to highlight what they liked about the programme, what they didn't like, and suggestions for change.

##### Demographics to be recorded

Self-reported socio-demographic variables will include age, gender, ethnicity, employment status, smoking & alcohol habits. Medical information will include co-morbidities and disease variables such as cancer grade, stage and regimen (chemotherapy, radiation therapy, hormonal therapy) will be collected from medical charts.

##### Additional details to be recorded

Protocol deviations, modifications made to any of the exercises will be recoded. Safety is paramount and any adverse events will be reported. We are aiming for a withdrawal and drop-out rate of < 10%. Protocol deviation such as switching or contamination, subjects dropping-out or adhering non-perfectly will also be recorded. The following details on compliance and adherence will also be recorded; compliance (X% of exercise sessions attended and successfully completed), adherence: we are aiming for adherence to exercise intervention (attendance or completion of exercise session) of > 70%.

### Exercise Training Interventions

The class will be supervised by a physiotherapist and a research assistant. There will be a maximum of 10 participants in each class, giving a ratio of at most 1:5 instructor: patient ratio.

The warm-up will consist of performing the mode of exercise prescribed for the work phase at a low intensity to allow the cardiopulmonary system to adapt to the new demand and to allow the temperature of the muscles to increase. Warm-up will be 7-10 minutes duration but slightly longer at the, initial stage of training.

#### Exercise intervention

The class will take 1 hour in total, with an aerobic component of 20-40 minutes increasing on a weekly basis (Table 1). The type of exercise will be continuous, rhythmic exercise using large muscle groups. There will be 3 aerobic stations: including treadmills, cycle ergometer, and rowing machine or stepper or other aerobic exercise such as marching on the spot deemed suitable by the class instructor.

**Table 1:** Progression of the intensity & duration of the exercise class

Intensity	Poor	Fair	Average
**Week 1**	35-55% HRR	40-60% HRR	45-65% HRR
	21 mins aerobic	21 mins aerobic	21 mins aerobic

**Week 2**	35-55% HRR	40-60% HRR	45-65% HRR
	24 mins aerobic	24 mins aerobic	24 mins aerobic

**Week 3**	40-60% HRR	45-65% HRR	50-70% HRR
	24 mins aerobic	24 mins aerobic	24 mins aerobic

**Week 4**	40-60% HRR	45-65% HRR	50-70% HRR
	30 mins aerobic	30 mins aerobic	30 mins aerobic

**Week 5**	45-60% HRR	50-65% HRR	55-70% HRR
	30 mins aerobic	30 mins aerobic	30 mins aerobic

**Week 6**	45-60% HRR	50-65% HRR	55-70% HRR
	36 mins aerobic	36 mins aerobic	36 mins aerobic

**Week 7**	50-60% HRR	55-70% HRR	65-75% HRR
	36 mins aerobic	36 mins aerobic	36 mins aerobic

**Week 8**	50-60% HRR	55-70% HRR	65-75% aerobic
	42 minutes aerobic	42 minutes aerobic	42 minutes aerobic

The class frequency will be twice per week and as well as exercise in the home environment, starting with at least 1 other day (alternating) exercise building up to 3 other days per week. Exercise in the home environment can be carried out in multiple sessions (but no less than 10 minutes each time). A polar watch will be worn during these exercise sessions to monitor intensity.

### Statistical analysis

Randomization will be by a computer-generated random numbers list (GraphPad Software, San Diego, CA) with intention-to-treat as the primary analysis and per protocol as the secondary analysis.

Assuming a difference of 5 mL/kg/min^-1^, with a SD of 6.5, and a 5% significance (two-sided) and 80% power, 28 participants would be required per group. However, the goal would be to accrue 32 subjects to allow for a 15% drop out rate and subgroup analyses. One year will be allowed for accrual of participants. The drop-out rate is calculated to include patients who are unfortunately diagnosed within 3 months of the final assessment as having a relapse of their cancer. As occult return of cancer could have a negative impact on fitness and capacity to exercise, these patients' data will be excluded from the primary outcome measure but included in the process analysis. The same will be done for the data of any participant on anti-Her2 therapy in whom an LVEF assessment during the study or within 3 months of the end of the study has a drop in LVEF to < 50% or by > 20% compared to baseline, as, similarly, occult ongoing myocardial toxicity could have a negative impact on fitness.

We will provide descriptive data and 95% CIs for all possible comparisons. For our primary analysis, we will use the intent-to-treat principle and for the secondary analysis, per-protocol analysis. In sensitivity analyses (intent-to-treat), the impact of missing values will be studied by using a range of imputed change scores for missing values, and by analysing those subjects who provide complete data. Repeated measured of analysis (ANOVA) will be used across more than two time points for continuous data. Mixed-model analysis will be used to model each outcome measure at three (or two) time points and compare the differences across groups over time for non-continuous data. We will use t-tests between 2 groups when data are normally distributed and the Mann-Whitney U test will be used when data are non-normally distributed. The Chi-squared test will be used for categorical data. Tests for interaction will be carried out for the effect of gender and type of cancer.

## Discussion

This study will assess the effect of a novel 8-week exercise programme in cancer survivors 2-6 months post cessation of adjuvant therapy. No intervention studies to date have explored a relatively short individually prescribed exercise intervention in a heterogeneous cancer population in terms of physiological and process outcomes. This study will provide valuable insights into the effect and feasibility of this type of programme and if successful would be clinically transferable to a broad range of cancer settings.

## Competing interests

The authors declare that they have no competing interests.

## Authors' contributions

JW, DOD and JH developed the idea for this study. JW was responsible for drafting the manuscript with contributions from DOD, JH and EG. EG will assess all subjects at the time-points outlined in the manuscript, and JW will implement the protocol and collect the data. All authors read and approved the final manuscript.

## Pre-publication history

The pre-publication history for this paper can be accessed here:

http://www.biomedcentral.com/1471-2407/10/42/prepub
